# Communication in high risk ante-natal consultations: a direct observational study of interactions between patients and obstetricians

**DOI:** 10.1186/s12884-020-03015-6

**Published:** 2020-08-27

**Authors:** Jo Hilder, Maria Stubbe, Lindsay Macdonald, Peter Abels, Anthony C. Dowell

**Affiliations:** 1 Department of Primary Health Care & General Practice, University of Otago, Wellington, New Zealand; 2 Department of Obstetrics and Gynaecology, University of Otago, Wellington, New Zealand

**Keywords:** Ante-natal clinic, Health provider – patient interaction, Health communication, Risk communication

## Abstract

**Background:**

Effective communication is crucial to any doctor-patient consultation, not least in pregnancy where the outcome affects more than one person. While higher levels of patient participation and shared decision making are recognised as desirable, there is little agreement on how best to achieve this. Most previous research in this area is based on reported data such as interviews or surveys and there is a need for more fine-grained analysis of authentic interaction. This study aimed to identify the discourse characteristics and patterns that exemplify effective communication practices in a high-risk ante-natal clinic.

**Methods:**

We video-recorded 20 consultations in a high-risk ante-natal clinic in a large New Zealand city with patients attending for the first time. Post-consultation interviews were conducted with the 20 patients and 13 obstetricians involved. Discourse analysis of the transcripts and videos of the consultations was conducted, in conjunction with thematic analysis of interview transcripts.

**Results:**

Most patients reported high quality communication and high levels of satisfaction; the detailed consultation analysis revealed a range of features likely to have contributed. On the clinician side, these included clear explanations, acknowledgement of the patient’s experience, consideration of patient wishes, and realistic and honest answers to patient questions. On the patient side, these included a high level of engagement with technical aspects of events and procedures, and appropriate questioning of obstetricians.

**Conclusions:**

This study has demonstrated the utility of combining direct observation of consultations with data from patient experience interviews to identify specific features of effective communication in routine obstetric ante-natal care. The findings are relevant to improvements needed in obstetric communication identified in the literature, especially in relation to handling psychosocial issues and conveying empathy, and may be useful to inform communication training for obstetricians. The presence of the unborn child may provide an added incentive for parents to develop their own health literacy and to be an active participant in the consultation on behalf of their child. The findings of this study can lay the groundwork for further, more detailed analysis of communication in ante-natal consultations.

## Background

Good communication is crucial to any doctor-patient relationship and has an important effect on patient outcomes including improved health, satisfaction, adherence to advice and information recall [1–3], not least in pregnancy where the outcome affects more than one person and the process and quality of ante-natal care will always be remembered as significant [4–6]. Maternity healthcare providers agree that listening to women and being empathetic are important, as is using effective non-verbal communication [7], especially when there are potentially negative outcomes [8, 9]. Women also value empathy, the opportunity and ability to ask questions, time, open and respectful communication, and informativeness [10–12]. It is also widely accepted that higher levels of patient participation and shared decision making in ante-natal consultations are desirable. However there is little agreement on how best to achieve this [11, 13], and women vary in how much information they want and how active or passive they prefer to be in antenatal consultations [14, 15].

It has been found that the clinical setting and clinician communication style have more influence on patient participation than patient attributes [3]. However, most studies investigating communication in maternity care have been restricted to aspects of communication in a narrow range of settings such as midwife consultations [16–20], genetic counselling [21–28] and women in labour [29–31], and research relevant to practitioners may be published outside the medical arena [32].

In addition to the need for more research on patient-provider communication generally in obstetric care [33], the specific details of communication style that are most effective in this setting remain under-investigated. Most studies investigating communication in maternity care rely on reported data such as interviews or surveys. Where observational methods are used (some with video- or audio-recordings), consultations are often coded using a deductive quantitative approach that does not take into account the subtleties of natural interaction [34–36]. Current advice for health professionals on how to improve communication therefore tends to be very general in nature, and does not take account of the complex, dynamic nature of interactions in real-life consultations, making it difficult for clinicians to implement in practice.

Whilst there is a substantial body of work involving analysis of directly observed interaction in primary care, particularly using video [37–44], there is much less research into the features of effective communication in other contexts. This is an important gap. For example, a study using discourse analysis in antenatal HIV/AIDS clinics in Malawi provided valuable insights into the use of humour as a communicative strategy [45]. However, there remains a need for more fine-grained interactional analysis to examine the detail of how participation and effective communication are manifested or facilitated in ante-natal consultations with obstetricians [21, 46].

Additionally, the overall structure of consultations and the sequencing of activities within them is an important aspect of effective communication in health contexts. Previous research [47] has analysed the structure of surgical appointments compared to those in primary care and identified a typically present “referral recognition sequence” (RRS) in which the specialist acknowledges the referral letter, including the reason for referral. This has been shown to be important for establishing a shared frame of reference and for the smooth progress of the consultation [47]**.** However, the structure and sequencing of specialist antenatal clinic consultations has not previously been investigated.

The aim of this study was to identify key discourse characteristics and patterns that exemplify effective communication practices in consultations in a high-risk ante-natal clinic by combining two types of data: direct evidence from consultation recordings, and post-consultation semi-structured interviews with participants.

## Methods

This qualitative study used mixed qualitative methods, drawing on the discourse analytic approach of interactional sociolinguistics [48, 49] and using multiple data sources. In contrast to much qualitative research in this area which often relies on a single type of data (frequently interviews), we combine direct observation and discourse analysis of verbal and non-verbal communication in video-recorded consultations with a thematic analysis of post-consultation interviews that focused on participant perceptions and experiences of communication in the same consultations.

Video recordings were made of routine consultations with consenting patients and obstetricians (either consultants^1^ or registrars^2^) in a high-risk ante-natal outpatient clinic in a tertiary hospital in a large New Zealand city. The hospital has a regional catchment that encompasses diverse socio-economic areas. Most pregnancies in New Zealand are primarily managed by publicly funded mid-wives or general practitioners, although some women choose privately funded obstetric care. These recorded consultations were undertaken in the public system where referral has come from a midwife or general practitioner in response to a specific indication of high risk. Referral letters are not normally given to patients in the New Zealand public health system.

Patients who were attending the clinic for the first time were asked for consent to participate. This was to ensure a more homogenous sample in that all patients would be likely to be meeting the clinician for the first time. Shortly after the consultation, short semi-structured interviews (mostly around 5 min) were conducted separately with both patients and obstetricians, and consent to obtain medical notes for the consultation was requested. The interviews used open questions to ask participants to comment on the communication in their consultation and their level of satisfaction (see appendix 1 for the interview guides used – in practice many of the questions to patients about risk were not asked due to the sensitivity of the topic and the time available).

### Data collection

Data was collected between June and November 2016. Consent from clinic staff to video some of their consultations was obtained ahead of time. Staff identified patients attending the clinic for the first time and a female researcher approached them in the waiting room to seek their informed consent (full briefing and written consent was then carried out in a private room). Where consent was granted, a single small camera on a slim tripod was discreetly set up in a corner of the consultation room so that the video captured all participants’ faces and most of their bodies. A small audio recorder was also placed on the desk as a backup. This equipment was set up and turned on at the beginning of the consultation by the researcher who then left the room. Interviews after the consultation were audio-recorded. The patient and any accompanying adults (often the woman’s partner) were asked for a brief interview in a consultation room immediately following the consultation. Obstetricians were interviewed at the end of their clinic, or by email if they preferred. Medical notes for the consultation were obtained where consent was given to support analysis and interpretation of the consultation recordings and interviews. The field researcher wrote ethnographic field notes to provide additional background information. Participants were also asked for consent for their data to be added to a permanent corpus of health interactions for potential future ethically approved research.

### Data analysis

The recordings of all consultations and interviews were fully transcribed and anonymised by the removal of identifying details such as names of people and places. A log of the activities in each consultation was created and proofed by a clinical member of the team. The inductive analysis followed an iterative process which alternated between individual analysis by the main field researcher (a discourse analyst and the team member with the most intimate knowledge of all the data) and group data sessions with the wider multi-disciplinary team of clinicians and applied linguists. The initial individual analysis combined a thematic analysis of the interview data (using NVivo software) in tandem with a structural discourse analysis of the consultations which identified macro-level features of the interactions including length of consultation, overall structure (including the RRS), how openings were managed and reasons for referral. This was supported by ethnographic information (field notes and medical notes). Validation (or otherwise) of these initial analyses was provided by the multi-disciplinary team which comprised an obstetrician, and general practitioner and a nurse and two non-clinical health communication researchers (experienced in interaction analysis and ethnography of communication). These data analysis sessions involved viewing and analysing video and transcript data against the analysis, and included attention to non-verbal features of the interaction including gaze direction, facial expressions, nods, body positioning and activities during silences. Later iterations of this two-stage process included searching for interactional evidence (or counter-evidence) in the consultation data for each of the themes emerging from the interview data. Any disagreements within the research team were resolved through discussion and consensus. Several further cycles of individual analysis and group data sessions resulted in a set of initial findings which were presented back to the clinical participants in the research, and the rest of the clinic staff. This provided an opportunity for further clinical feedback on our methods and findings, which then led to further revision and refinement.

All analysis deliberately followed an appreciative enquiry approach [50–52]. The focus was thus principally on identifying positive features of the interaction as well as locating interactional evidence for the themes from the interviews which represented the participants’ perpectives. At the same time, other relevant features that came to light were noted and taken into account in the analysis and interpretation of the data.

Patients were not involved in the design or analysis of the study.

## Results

### Participants

Thirty two patients were approached to participate, of whom 7 declined. A further 5 patient consultations were not recorded for logistic reasons. Recordings were made of 20 patients in consultations, 11 of whom were accompanied by a partner or other family member (18 video and 2 audio only). One consultation was only partially recorded at the doctor’s request. Sixteen of the 20 patients and those accompanying them were interviewed (the remaining patients declined due to time constraints). Two patients declined consent for their medical notes to be obtained. Thirteen obstetricians were filmed across the 20 consultations, all of whom were interviewed afterwards (one via email).

### Participant characteristics

The patients were between 20 and 39 years old, with more in the older age bracket (12/20 aged 30–39); half were of European background (most of whom were New Zealand born) and most were well-educated. More than half (13/20) had given birth before. (See Table 1).

**Table 1:** Demographic characteristics of patients

	***n*** = 20
**Ethnicity**	
NZ European	8
European	2
Māori	2
Pacific	2
Asian	6
**Age**	
20–24	1
25–29	7
30–34	5
35–39	7
**Education**	
PhD	1
Masters	2
Bachelors	4
Polytechnic degree	9
Professional Qualification	1
Secondary School	2
Other	1
**Parity**	
0	7
1	10
2	1
3	2

The 13 obstetricians in the study included 6 consultants (11 consultations recorded) and 7 registrars (9 consultations recorded). Most were female (9/13) including all of the registrars, and included overseas and locally trained doctors with a range of experience.

### Consultation characteristics

As background to and context for the analysis of the quality of the communication that follows, we initially report on several descriptive features of the consultations: length, structure, reasons for referral and the structure of the consultation openings.

### Length of consultations

The length of consultations ranged from about 15 min to nearly an hour. Of the 19 full length consultations recorded, 13 were 15–30 min in length and six were 30–60 min. The average length of consultation was 28 min, with registrars being more likely to have longer consultations.

### Consultation structure

Eleven of the consultations began with a discussion of the main referral issues while six began with the medical history (three had an unclear structure). There was a tendency for the more experienced obstetricians to deal with the main referral issue first and to back-fill the medical history later in the consultation, while registrars most often began with the medical history.

Most of the doctors (17) read the patient notes prior to beginning the consultation, although for three of the patients who saw a consultant, the notes were read during the consultation.

### Reasons for referral

The reasons for referral to the high-risk antenatal clinic were varied, and often there was more than one reason for referral. The most common reasons were previous caesarean delivery - often in combination with other reasons (7), large or small for dates (2), twins (2) and bleeding (3). Other reasons for referral were: recurrent miscarriage; high BMI^3^; threatened pre-term labour; previous infant deaths; hip dysplasia; recurrent herpes; low platelets; episode of dizziness/shortness of breath; low thyroid levels; schizophrenia management; malignant hyperthermia; latent tuberculosis; and previous HLH^4^ infant deaths.

### Referral recognition sequences

A clear referral recognition sequence (RRS) was identified in 18 of the 20 consultations.^5^ Table 2 shows the patterns for referral recognition sequence (RRS) that were found.

**Table 2:** Patterns of Referral Recognition Sequence (RRS)

Type of RRS	Example	N
1. *Immediate explicit RRS*		9
a. ‘who from’ and ‘why’	“So your midwife … has asked you to come in and see us today because baby’s a little bit small?” (AN-SP25R-01)	5
b. Immediate explicit RRS ‘who from’ with delayed ‘why’	SP: “So you’ve been referred to us by your midwife right?” … [SP reads notes] PT: “So what is this appointment about today?” (AN-SP29–01)	4
2. *Immediate implicit RRS*	“So because this is your first visit … I’ll ask you a few questions about yourself and then we’ll talk about the twins” (AN-SP28R-01)	6
3. *Elicitation of patient perspective*		3
a. Immediate explicit RRS (‘who from’ and/or ‘why’) plus elicitation of PT perspective	*“*So the reason that you’ve been referred was that I understand that you had a bit of bleeding when you were in ((COUNTRY)). Okay, tell me a little bit about that” *(*AN-SP28R-02)	1
b. Immediate elicitation of ‘why’ (with or without ‘who from’)	“So I guess the first thing is, do you know why you’re here?” (AN-SP36R-01)	2
	“alright so i’ve got a referral for you from the midwife just telling me a wee bit about you know why you’re here? obviously you guys are pregnant congratulations um, but, yeah do you wanna sort of tell me in your own words what’s sort of what’s been happening and where you’re at and, why you’re here” (AN-SP27–02)	

In half of the consultations, the RRS was explicit and mentioned both who had made the referral and why, although in nearly half of these, there was some delay in relaying the reason for referral. This delay meant that patients were unclear about some aspects of the referral for a time and could result in some discomfort where patients explicitly asked for the reason for their referral (as in the example for RRS Type 1b), or in other cases showed increased anxiety. This happened when patient notes were being read during the consultation, which was more common among the more experienced consultants. Apart from this tendency, there were no other clear differences between consultants and registrars in the use of RRS patterns.

### Quality of the communication in the consultation

Analysis of participant interviews and of the consultation recordings highlighted a number of features that contributed to a sense of high quality communication and of patient satisfaction. Patient interview responses were almost universally positive, with all but one of the patients expressing satisfaction overall with the communication in the consultations. Comments on communication ranged from “good” (AN-SP34R-01 PT interview), to “he’s very good with his communication skills” (AN-SP29–01 PT interview), through to “amazing…. I think the best doctor I’ve seen.” (AN-SP36R-01 PT interview).

The following more specific positive features of the communication emerged from the mixed methods analysis. Tables 3 and 4 contain illustrative quotes from the interview data and excerpts from transcripts of the actual consultations that are referred to below.

**Table 3:** Interview quotations that illustrate the themes from the interactional analysis

**1 Informative, thorough and clear communication**	
*Quote 1.1:* “you could see what was going on in their head a little bit” AN-SP25R-01 (PA) (PT interview) *Quote 1.2:* “For a person that I haven’t met before, [they were] really good with explaining information…it’s hard to find doctors who can actually sit you down and take you through everything.”^6^ AN-SP29–01 (PT Interview) *Quote 1.3:* “Yeah, I mean [they were] very thorough” AN-SP26–02 (PT Interview) *Quote 1.4:* “[they] definitely addressed everything that we needed to think about.” AN-SP30R01 (PT Interview)	
**2 Explanations delivered in clearly signalled components**	
*Quote 2.1:* “The way they structure it... instead of jumping from one information to the other, [they] must have structured the whole entire appointment, yeah, which was really good.” AN-SP29–01 (PT Interview) *Quote 2.2:* “talked point by point... rather than looking at the big, scary picture. Sort of broke it down, which made it a lot easier to be able to discuss you know, the little details, and then building up into the... the big conversations” AN-SP36R-01 (PT Interview)	
**3. Feeling listened to and their experience acknowledged (especially anxiety)**	
*Quote 3.1:* “they didn’t just cut me off and dismiss my... concerns” AN-SP34R-02 (PT Interview) *Quote 3.2:* “it felt like it’s okay to be worried kind of you know, like it’s okay how you’re feeling” AN-SP25R-01 (PT Interview)	
**4. Feeling able to ask questions and get them answered**	
*Quote 4.1:* “Lots of opportunity to ask questions” AN-SP30R-01 (PT Interview) *Quote 4.2:* “I did ask a lot of questions…. but they were all answered, so yeah.” AN-SP31–02 (PT Interview)	
**5. Consideration of patient wishes and provision of options**	
*Quote 5.1:* “[they] gave us options” AN-SP27–02 (PT Interview) *Quote 5.2:* “Just like, ask me my opinion, what I want to do” AN-SP31–01 (PT Interview)	
**6. Realistic and honest communication**	
*Quote 6.1:* “[they are] quite realistic, so that’s something I like to know. I don’t want to bat around the bridges.” AN-SP29–01 (PT Interview) *Quote 6.2:* “it felt it felt very open … and kind of factual and that they weren’t hiding anything i suppose” AN-SP25R-01 (PT Interview)	
**7. Good rapport**	
*Quote 7.1:* “[they] made me feel very at ease pretty quickly … [they] seemed very relaxed, and … I guess it made me more relaxed too” AN-SP36R-01 (PT interview) *Quote 7.2:* “[they] made me feel very comfortable” AN-SP28R-02 (PT interview)	
**8. Patient displays of knowledge**	
*Quote 8.1:* “I think she came in with a good idea about these risks.” AN-SP29–01 (SP Interview) *Quote 8.2:* “They were a lovely couple that had already done a lot of reading... which makes it a lot easier. I think it’s difficult when people come in and they have either done no reading, or have no idea about which way they want to go. And then it becomes a lot more difficult, because of the clinic... we do kind of pressure them into trying to make a decision one way or the other. But then that is difficult to make such a big decision in a sort of 15 to 30-min consult. So they were sort of already well up-to-date with what they needed to know. So that was helpful.” AN-SP30R-01 (SP Interview; VBAC clinic) *Quote 8.3:* “I think they’d come well prepared” AN-SP29–03 (SP Interview)	

6Note, pronouns referring to obstetricians have been changed to “they” to further protect anonymity.

**Table 4:**
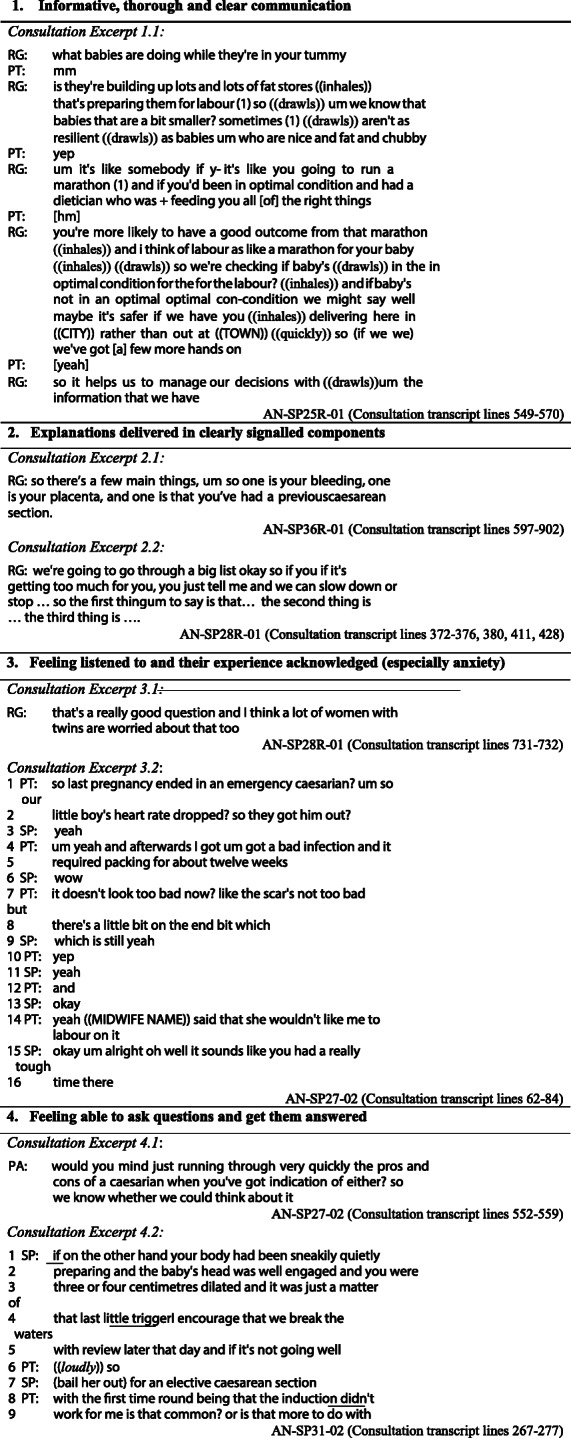

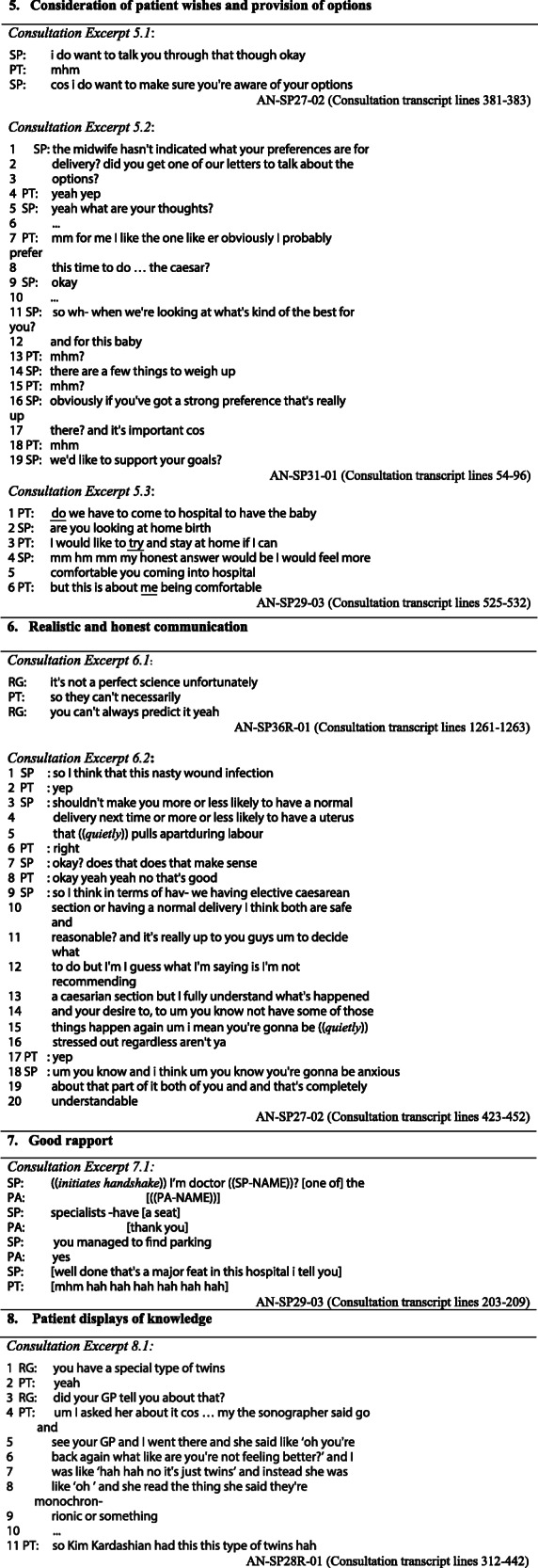
Data extracts from the consultation transcripts that illustrate the interactional analysis

1. *Informative, thorough and clear communication*

Nearly every patient (and/or accompanying adult) who was interviewed (15/16) mentioned this in their evaluations of the communication, as shown in the selection of quotes in Table 3. One patient appreciated the way in which the thought processes of the doctor were made visible (Quote 1.1), while another specifically acknowledged the difficulty of good communication with someone you have not met before (Quote 1.2). Patients also explicitly mentioned thoroughness or described a thorough approach, as in Quotes 1.3 and 1.4. Within the consultations, this was evidenced by the coverage of multiple topics and the detailed discussions observed (the length of which make it impractical to reproduce an example here). An example of clear and informative communication is given in Consultation Excerpt 1.1 in which the registrar goes to some length to explain the reason for the extra concern with a baby that is small for dates, using an extended metaphor to make the point.

2. *Explanations delivered in clearly signalled components*

Informativeness and clarity was enhanced by clearly structured communication that patients reported experiencing in their consultations, as evidenced in Quotes 2.1 and 2.2.

Clear signalling of topics and agendas was directly observed in many of the consultations; this made the structure more obvious, as seen in Consultation Excerpts 2.1 and 2.2 in which the doctor first explicitly indicates they are about to deliver an ‘informing’, then numbers off the topics to be discussed in the consultation as a way of signposting the stages of the explanation.

3. *Feeling listened to and feelings/experience acknowledged (especially anxiety)*

While the question was not always specifically asked in the semi-structured interview (depending on how the conversation went), when 12 patients were explicitly asked if they felt they were listened to, all responded positively for example, in Quote 3.1. One patient in particular mentioned feeling that their anxiety had been legitimised (Quote 3.2).

Within the consultation, several doctors explicitly acknowledged the worry or anxiety patients may feel, as shown in Consultation Excerpt 3.1, and also in excerpt 6.2 (lines 13–20). In excerpt 6.2 (lines 15–16), the clinician explicitly claims to understand that the patient is and will be ‘anxious’ and ‘stressed out’, using informal language (such as ‘gonna’ and ‘aren’t ya’) that serve to minimise the social distance between clinician and patient, and quiet talk to index the sensitivity of this. The clinician also phrases their statement so that patient agreement is the ‘preferred response’ [53] by using a tag question (‘aren’t ya’). The patient does indeed agree (line 17).

A good example of a patient’s previous experience being acknowledged with empathy is shown in Consultation Excerpt 3.2, where in addition to the minimal responses (such as “yeah”), the doctor provides brief but effective acknowledgement of the impact of the experience on the patient at line 6 with a simple “wow”, and with an explicitly empathic statement at line 15–16.

4. *Feeling able to ask questions and get them answered*

Many patients (9/16) specifically mentioned in interviews that they felt comfortable to seek further information or explanation, as seen in Quotes 4.1 and 4.2. One illustration is seen in Consultation Excerpt 4.1 where the partner of the patient spontaneously asked the doctor to provide more information, which was responded to at length.

In Consultation Excerpt 4.2, the consultant has been giving information at some length, and it is noteworthy that the patient, at line 6, begins an assertive bid to participate (“so”, just before the consultant has finished speaking. The patient here successfully gains the floor at line 8 and asks their question. This illustrates that even in more challenging interactional contexts such as this, where a specialist is engaged in an extended informing sequence (which patients typically do not interrupt), the patient here indeed had a level of comfort with active participation. The doctor, while completing their turn in the face of the patient’s bid for a turn, then gives the floor to the patient, maintaining mutual gaze and nodding as a ‘go-ahead’.

5. *Consideration of patient wishes and provision of options*

Five of the patients specifically mentioned the fact that they were given options and that they felt their wishes were sought and respected, as seen in Quotes 5.1 and 5.2. Again there was evidence of this occurring in the consultations. Consultation Excerpt 5.1 illustrates an obstetrician explicitly telling patients that they aim to inform them of their options. Consultation Excerpt 5.2 is from a Vaginal Birth After Caesarean (VBAC) clinic consultation with a non-native speaker of English. The clinician makes it clear that patients’ preferences for VBAC or caesarean will be considered, quickly checking on whether the patient has received information on the options. At line 5, the clinician asks a completely open question that is not tilted towards either of the available options. The patient expresses her preference for a caesarean section and the clinician explicitly affirms the importance of the patient’s preferences in lines 16–19, after noting the need for clinical assessments (lines 11–14).

In another consultation (for which no patient interview was conducted), there was a little more negotiation as to whose wishes might prevail (see Consultation Excerpt 5.3). When the obstetrician expressed an opinion that was at odds with the patient’s preferences (lines 4–5), there was push-back from the patient in line 6. The obstetrician went on to explain the risks if a herpes lesion was present in labour and that the patient may not be aware of a lesion, adding:

“as long as you're aware of that situation then you'd be better informed to make that decision, that's number one”

While emphasising the importance of medication and extra scans regarding small gestational size, the obstetrician also acknowledged that the patient had a “fair point” on several occasions during the consultation, which explicitly validated the patient’s perspective. By presenting information and options and acknowledging the patient’s perspective, even when being challenged, the obstetrician succeeded in keeping the interaction on positive terms and negotiations friendly and respectful.

6. *Realistic and honest communication*

Two patients particularly appreciated straightforward and realistic communication from the doctors (Quotes 6.1 and 6.2). Other patients reported positively on consultations in which open and realistic talk was observed, such as Consultation Excerpt 6.1 in which a registrar comments on the inherent uncertainty in this setting. Consultation Excerpt 6.2 is another example of plain talking that appeared to be appreciated. In line 5, the doctor uses very direct, colloquial language to talk about the possibility of the uterus ‘pulling apart’, albeit softening the words by lowering the volume of talk.

7. *Good rapport*

Several patients mentioned the way in which doctors made them feel relaxed and comfortable, as in Quotes 7.1 and 7.2.

Simple things like handshakes and small talk that may elicit laughter contributed to building rapport and making patients and those accompanying them feel welcome and comfortable. A simple example is shown in Consultation Excerpt 7.1 where the patient’s partner, who arrived late, was explicitly made welcome with introductions and small talk that elicited laughter.

8. *Patient displays of knowledge*

In addition to the opportunities patients had to ask questions and participate, patients and those accompanying them were also able to display their knowledge, with many of them having experienced childbirth before. Such patients spontaneously used clinically appropriate technical terms such as “placenta praevia” (AN-SP31–02) or “breech” (AN-SP36R-01). The patient in Consultation Excerpt 4.2 (discussed above) displayed her confidence in her knowledge with an interruption (line 6) that treats the doctor’s partial utterance (‘if it’s not going well’) as sufficient (indicating that she doesn’t need to hear the rest of the explanation). Her following turn (lines 8–9) further displays her understanding by her use of the term ‘induction’ in a way that links back to the doctor’s discussion of triggering labour by breaking waters (i.e. displaying her understanding that this is a form of induction).

In interviews, the obstetricians explicitly valued patients being well-informed, especially in view of the limited consultation time available (see Quotes 8.1–8.3), and were observed in consultations giving patients opportunities to display their knowledge, thus also ascertaining their current understanding. Even those without previous childbirth experience were given opportunities to display recently acquired knowledge, as shown in Consultation Excerpt 8.1 in which the patient is a young first time mother. Here the doctor initially talked in non-technical terms (line 1) and asked a question at line 3 which opened up the floor to the patient to answer with a narrative that led up to her attempting to provide the technical term herself. The patient also later displayed her familiarity with the type of twins in line 11.

## Discussion

### Main findings

This study explores the quality of communication in routine obstetric ante-natal care using methodology that combines direct observation techniques with experiential data from patient interviews, and with a specific focus on identifying specific features of consultation discourse that contributed to patient-reported satisfaction and quality of communication.

Overall, patients’ reports on the communication in their consultations were very positive, and these reports were confirmed by researchers’ analysis of the recordings which indicates that the clinicians in this setting were able to meet women’s need for effective communication [54]. Some of the consultations were quite long (up to 1 hour), and the length of consultations may have contributed to high satisfaction levels.

The overall structure of the consultations varied according to the level of experience of the doctors, with more experienced consultants able to “cut to the chase” by not exhaustively following medical checklists. While most of the existing literature about consultation length comes from General Practice [55], this study aligns with hospital outpatient findings in which registrars took more time with patients than consultants [56]. Registrars sometimes completed a medical history before moving to the referral and this may have contributed to their longer consultations. The importance of the opening of the consultation and of the referral recognition sequence (RRS) in specialist practice, as reported elsewhere, was confirmed in this data set; when there were delays in the full RRS being completed, the smooth progression of the consultation was disrupted with patients sometimes asking explicitly why they were there or showing signs of increased anxiety in the face of this uncertainty.

Generally patients reported that their feelings were heard, anxieties acknowledged and questions answered. Again this was reflected in observations of the recorded consultations, which also showed high levels of participation and invitations for shared decision making. Many patients displayed fluency in many aspects of the discussion in the consultations, including familiarity with clinical terminology and a biomedical framing of the conditions contributing to the high risk state, especially where they had previous experience of complications in pregnancy and childbirth. This is not to say that the communication observed was uniformly positive, but our appreciative inquiry stance provides a constructive basis for recommending strategies and practices that are likely to enhance quality of communication.

### Strengths and limitations

We are not aware of other studies that have directly observed the detailed interactional processes at work in a generic high risk ante-natal clinic, and that correlate these direct observations with interview data from both patients and clinicians. Limitations of the study are the small size of the data set, possible skewing in the patient sample towards older and more highly educated women, and the observer effect (the communication in the consultations may have been affected by the presence of the recording devices, with participants perhaps inclined to show themselves in the best light). The interviews were also limited by their short length (to minimise imposition on participants). Some patient responses in interviews may have been affected by a reluctance to criticise their clinicians.

### Interpretation

Our results show that communication in the high risk ante-natal context studied here differs from that documented in other health settings in a number of ways, notably the high levels of patient participation and the ways in which risk is discussed.

Given the challenges that clinicians in all disciplines often face in achieving effective interaction with patients, the results portray a positive picture of doctor patient communication and a relatively empowered and well-informed cohort of patients [57–59]. Our results accord with survey studies that found an association between ‘high quality’ interactions with realistic and clear information and reassurance [60, 61]. The high level of patient satisfaction is consistent with a 2008 review of maternity services in this region of New Zealand which found low levels of dissatisfaction among 115 participants [62]. The specific elements that were highly rated in our interviews and observed in the consultation data are largely consistent with more high-level descriptions in other studies of what patients want from maternity care generally [10–12, 54].

Previous research on surgeon communication has emphasised the need to make sure that patients are enabled to present their problems fully, despite the surgeon already having another source of information (achieved through the RRS) [47]. This ensures that patient and doctor are aligned about the purpose of the consultation. Attending to this by getting the topic of ‘risk’ on the table early in the consultation is also a way to reduce anxiety for patients who, by definition, know they have been referred for some reason. Our analysis of the ways in which the specialists in our data achieve the referral recognition sequence provides detail about the communication that underpins overall perceptions of effective communication, and this can inform training in medical communication.

The high level of patient participation observed in this study contrasts with earlier studies which found that many women undergoing antenatal care did not actively participate and were uncomfortable with decision making responsibility [15], rarely asked questions and were not encouraged to do so [63], and often did not share in decision making [34]. The increased ‘agency’ that we observed may be due to recent efforts to develop more ‘patient-centred’ care [64–66] and shared decision making [67, 68]. The high participation levels may also have been influenced by the patients feeling that they were being given options and their choices respected, an endorsement of the communication styles of the study doctors. The fact that patients felt that the doctors were informative, clear and realistic with the information they provided also contributes to shared decision making.

Ante-natal and maternity care is a clinical environment where health literacy can have an important impact on outcomes [69]. We observed examples where patients displayed knowledge and were willing to ask questions in often complex areas of care such as choices regarding operative intervention or the type and timing of imaging, indicative of high levels of health literacy. The presence of the unborn child as an unseen and unheard additional patient who needs to be cared for by the mother (and partner) may provide an added incentive for patients to further develop their own health literacy and to be an active participant in the consultation on behalf of their child.

The explicit way that risk is talked about was noticeable in the interactions and reported by patients as being appreciated. Risk was framed in ways that were clearly articulated and understood. Explicit orientation to risk seems acceptable in this context where patients have been referred to a high risk antenatal clinic, a finding that is congruent with other research [70]. For example, in this study doctors opted to manage the potential for causing anxiety by explicitly addressing clinical risks, rather than avoiding such talk which may then prolong uncertainties and worry [70]. Focus group studies with women on topics such as gestational weight gain also indicate that women prefer these issues to be addressed explicitly but sensitively [71–74].

## Conclusion

This study provides detailed information about communication in ante-natal care, and has identified a number of features of interaction in consultations which may explain high levels of satisfaction by patients (and those accompanying them). In contrast to much of the literature which emphasises the challenges of appropriate communication and shared decision making in maternity care, this case study has provided many examples of good communication practice. Previous research has identified a need for better training in obstetric communication [75], especially in handling psychosocial issues and in conveying empathy, which have been shown to be teachable [76, 77]. The findings of this study can inform communication training for medical students and other less experienced health professionals and lay the groundwork for further, more detailed analysis of such communication.

## Appendix 1:Interview guides for patients and clinicians

**Interview guide - Patients.**

Preamble. Thank you again for agreeing to be part of this study. I’m going to ask you now about the consultation you have just had. We are especially interested in communication; in how information is exchanged between patients and doctors.

1. Have you met this doctor before?

2. Is this your 1st pregnancy? (specify if not).

3. Tell me briefly what this consultation has been about.

I would like to ask you now a bit more about your pregnancy and how you think things are going. CHOOSE FROM FOLLOWING AS APPROPRIATE FOLLOWING INITIAL CONVERSATION

- Has the doctor indicated there are any things to be looking out for in your pregnancy, or any things that are going to be monitored or checked during the pregnancy?
- How do you feel about those things?
- Do you think they make your pregnancy more risky than it might be otherwise?
- Has the doctor talked to you about risks?
- What do you understand about those risks?
- Overall do you think there are particular things which make your pregnancy more risky than in your previous pregnancy (or compared to other women having a baby if this is the first pregnancy)
- Do you think these risks are mainly to the baby and or to you?
- In what way?
- Do you think the risks would be mainly affecting you / your baby before, during or after the baby’s birth?
- How well do you think the doctor explained those risks to you?
- Do you feel reassured about things as a result of what the doctor has said?
- Are there any other things that have reassured you or that would reassure you?

4. Did you understand everything that the doctor talked about?

5. Do you think the doctor listened to you and heard what you had to say?

6. Are there any other challenges or difficulties?

7. What other comments would you like to make about communication in this setting?

8. Were you satisfied with the outcome of the consultation?

9. Do you think the video recorder being there changed the communication in any way?

**Interview guide - Clinicians**

1. Quick summary of content and outcome of the consult
2. Any comments on how the communication went (any challenges or why it went particularly well or not)?
3. Do you think the video recorder being there changed the communication in any way?

## Abbreviations

RRS: Referral recognition sequence
VBAC: Vaginal Birth After Caesarean

## Key to transcription

SP: Specialist
RG: Registrar
PT: Patient
PA: Patient’s partner
[ ]: indicates simultaneous speech
more: emphasis
((*loudly*)): indicates how the following words in italics are spoken; or non-verbals
((NAME)): name removed for confidentiality
(1): pause of 1 second

## Footnotes

1 i.e. fully trained specialists (‘attending physician’ in the USA)

2 i.e. specialists in training/under supervision

3 Body Mass Index

4 Hemophagocytic Lymphohistiocytosis

5 The lack of RRS in the remaining 2 consultations was for clear reasons: one patient, while technically new to the clinic, was actually continuing care with the same obstetrician; the other was attending a follow-up to an emergency hospital admission.
